# Error Propagation in the Simulation of Atherosclerotic Plaque Growth and the Prediction of Atherosclerotic Disease Progression

**DOI:** 10.3390/diagnostics11122306

**Published:** 2021-12-08

**Authors:** Antonis I. Sakellarios, Panagiotis Siogkas, Vassiliki Kigka, Panagiota Tsompou, Dimitrios Pleouras, Savvas Kyriakidis, Georgia Karanasiou, Gualtiero Pelosi, Sotirios Nikopoulos, Katerina K. Naka, Silvia Rocchiccioli, Lampros K. Michalis, Dimitrios I. Fotiadis

**Affiliations:** 1 Department of Biomedical Research, Institute of Molecular Biology and Biotechnology—FORTH, University Campus of Ioannina, 45110 Ioannina, Greece; psiogkas4454@gmail.com (P.S.); kigkavaso@gmail.com (V.K.); panagiota.tsompou@gmail.com (P.T.); savvasik21@gmail.com (S.K.); g.karanasiou@gmail.com (G.K.); fotiadis@uoi.gr (D.I.F.); 2 Unit of Medical Technology and Intelligent Information Systems, Department of Materials Science and Engineering, University of Ioannina, 45110 Ioannina, Greece; dipleouras@gmail.com; 3 Institute of Clinical Physiology, National Research Council, 56124 Pisa, Italy; pelosi@ifc.cnr.it (G.P.); silvia.rocchiccioli@ifc.cnr.it (S.R.); 4 Department of Cardiology, Medical School, University of Ioannina, 45110 Ioannina, Greece; soter1313@yahoo.gr (S.N.); drkknaka@gmail.com (K.K.N.); lamprosmihalis@gmail.com (L.K.M.)

**Keywords:** coronary artery disease (CAD), error propagation, computational modeling, 3D reconstruction, plaque growth, predictive models

## Abstract

Assessments of coronary artery disease can be achieved using non-invasive computed tomography coronary angiography (CTCA). CTCA can be further used for the 3D reconstruction of the coronary arteries and the development of computational models. However, image acquisition and arterial reconstruction introduce an error which can be propagated, affecting the computational results and the accuracy of diagnostic and prognostic models. In this work, we investigate the effect of an imaging error, propagated to a diagnostic index calculated using computational modelling of blood flow and then to prognostic models based on plaque growth modelling or binary logistic predictive modelling. The analysis was performed utilizing data from 20 patients collected at two time points with interscan period of six years. The collected data includes clinical and risk factors, biological and biohumoral data, and CTCA imaging. The results demonstrated that the error propagated and may have significantly affected some of the final outcomes. The calculated propagated error seemed to be minor for shear stress, but was major for some variables of the plaque growth model. In parallel, in the current analysis SmartFFR was not considerably affected, with the limitation of only one case located into the gray zone.

## 1. Introduction

Cardiovascular disease (CVD) is the most common cause of death being responsible for significant escalating healthcare costs [1]. The main pathology of CVD is atherosclerotic disease, which develops with slow rates during the lifetime, but its events are usually acute, causing death if medical care is not provided on time [2]. The assessment of CVD is achieved using invasive and non-invasive imaging. The gold standard is still considered invasive X-ray coronary angiography, but computed tomography coronary angiography (CTCA) has recently been proposed by the European Society of Cardiology to be used in clinical practice, as the first non-invasive diagnostic imaging method [3].

Preventive and predictive strategies have been developed in recent years utilizing datasets which include patients’ information and follow-up outcomes. Traditional approaches are based on statistical models, while the most recent are based on machine learning algorithms. Moreover, the use of coronary imaging enables the 3D reconstruction of the coronary arteries and their utilization for the computational simulation of blood flow towards the estimation of endothelial shear stress (ESS), since it has been found that low ESS is an independent predictor of disease progression [4,5,6]. Furthermore, advanced computational models simulating the main mechanisms of atherosclerotic disease evolution and growth also exist [7,8].

The accuracy of the computational models of blood flow or plaque growth depends mainly on the accuracy of the reconstructed coronary arteries. In a previous error propagation study [9], the imaging acquisition included some errors which propagated in the analysis of the images, the segmentation of the lumen and the outer wall borders and plaque characterization. In parallel, the segmentation algorithms and reconstruction methodologies include additional error, which may in turn increase significantly the acquisition error. Thus, it is expected that this imaging-based propagated error should affect the computational results and the accuracy of the predictive models.

In this work, we present, for the first time, the effect of the imaging based propagated error on the computational results and the accuracy of the developed predictive models. In particular, through the utilization of 3D coronary arteries with and without error, we employed previously developed models of blood flow, fractional flow reserve (FFR) calculation and plaque growth to estimate the propagated error. The propagated error was calculated for each level of analysis (3D reconstruction, blood flow modelling and plaque growth modelling). Moreover, an uncertainty was calculated considering the error of the previous levels. Moreover, we develop predictive models of disease progression to investigate the degree that the propagated error affects accuracy.

## 2. Materials and Methods

### 2.1. Study Population and 3D Reconstruction

Twenty patients were recruited from the SMARTool clinical study (ClinicalTrials.gov Identifier: NCT04448691) [10]. For each patient, baseline and follow-up clinical, biohumoral data and CTCA imaging data (mean follow-up period: six years) were collected. Full explanation of the investigational nature of the study was provided to all participants and written consent was obtained. Ethical approval was provided by each participating center (National Research Council, Pisa Italy, University of Turku, Turku, Finland, University of Zurich, Zurich, Switzerland, Fondazione Toscana Gabriele Monasterio, Pisa, Italy, Warsaw National Institute of Cardiology, Warsaw, Poland) through the approval of the clinical study by the Ethics Committee Vast Area Northwest of Tuscany (CEAVNO), Pisa, Italy, and all subjects gave written informed consent. Our clinical study follows the declaration of Helsinki.

The 3D reconstructions of the coronary lumen and outer vessel walls were performed using an in-house software, which provided measurements of lumen area, plaque area and plaque burden, as previously described and validated by IVUS-VH and manual annotations [11].

### 2.2. Error Generation

**Figure 1:**
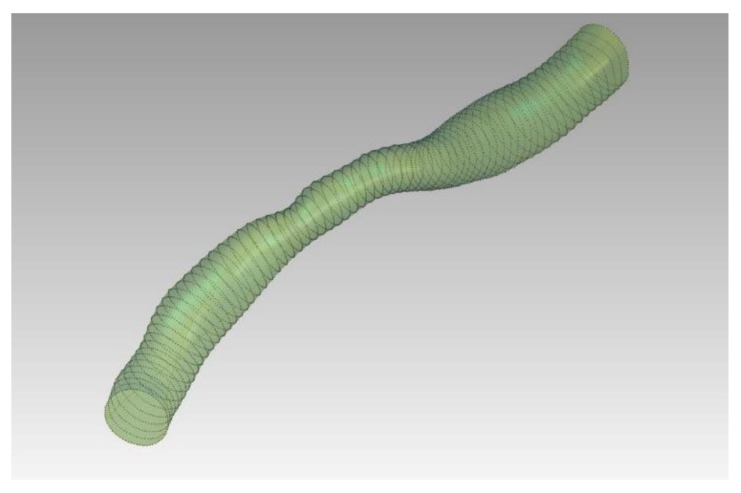
Illustration of a 3D model along with its respective contours in point cloud format.

To create the error propagation models, we made a random selection of twenty patients who were either healthy or had an ischemic incidence (i.e., pathologic fractional flow reserve (FFR) or significant stenosis > 50%). For each reconstructed vessel, apart from the two 3D volumes that were automatically created by the reconstruction module (i.e., the lumen and outer wall), two models with equidistant contours in point cloud format (Figure 1) were also included.

Each contour consisted of 72 points. The process of the error-containing 3D models is presented below:

- In order to increase or decrease the lumen area for each contour, the centroid of the contour was calculated.
- The original points of the contour were then scaled according to the desired area reduction factor. In our case we determined an error of 5% in each contour area.
- The centroid of the scaled contour was then determined.
- The translation vector was then calculated by finding the difference of the two centroids.
- The translation vector was used to find the translation between the original points and the scaled ones.
- The translation was applied to the scaled points, thus creating the scaled contour.
- The overestimated and the underestimated models were then created by converting the point clouds to 3D volumes.
- The error of 5% was determined using the validation results of the 3D reconstruction algorithm [11,12].

The same procedure was followed for the outer wall, as well. This resulted in 60 arterial segments in total.

### 2.3. Blood Flow Modelling and SmartFFR Calculation

The Navier–Stokes equations were employed for the simulation of the blood flow, assuming that the flow is laminar and incompressible. The blood was defined as a Newtonian fluid, having a viscosity of 0.0035 Pa s. For the error propagation analysis of ESS values, the results produced for a flow rate of 1 mL/s, which corresponds to the average blood flow during rest, were applied as boundary condition.

SmartFFR [13,14], which is a computational approach to estimate invasive FFR, was calculated based on a transient simulation of blood flow (consisting of 5 timesteps with timestep duration 0.25 s), applying an average pressure 100 mmHg at the inlet (i.e., mean human aortic pressure) and, at the outlet, a volumetric flow rate 0, 1, 2, 3 and 4 mL/s was applied per timestep. No-slip and no-penetration boundary conditions were used at the endothelial membrane boundary. In the next step, for each timestep, the Pd/Pa value was calculated in order to create a Pd/Pa curve using a spline of 100 points. The area under this curve is calculated and normalized using the area under curve of a healthy segment. This ratio represents the SmartFFR value [15].

### 2.4. Plaque Growth Modelling

A previously developed plaque growth model was used for the simulation of the growth of atherosclerotic plaque in the arterial wall [8]. Using this model, the user can simulate a time-dependent wall thickening and the gradual lumen narrowing and blockage of blood flow. Briefly, the model was based on the modelling of blood flow in the lumen and the transport of low density lipoprotein (LDL) [5], high density lipoprotein (HDL) and monocytes in the lumen, employing convection-diffusion equations. The endothelial membrane acts as a barrier between the lumen and the arterial wall and we applied the Kedem–Katchalsky equations to define the path of molecular penetration in the wall. In parallel, differential equations are employed in the arterial wall to simulate the LDL oxidation, the initiation of inflammation by the transformation of monocytes into macrophages, foam cell formation and the proliferation of smooth muscle cells (SMCs) and collagen production. At the final step, we simulate the time-dependent plaque volume which is assumed to be comprised by SMCs, foam cells and collagen.

In the current study of error propagation, we assume that the reconstruction error affects the plaque growth results. The assumption is based on the following considerations: (i) the endothelial permeability depends on the local ESS. In that sense, the error of the reconstructed geometries is propagated to the calculation of the ESS through blood flow modelling, which in turn, increases or decreases endothelial permeability, which may change the progression rate of plaque. (ii) The reconstruction of the outer wall may affect the accumulation rate of the molecules in the arterial wall due to the altered pressure difference between the adventitia and the endothelium boundary. (iii) In the plaque growth model, we assume some initial concentrations of the SMCs in the arterial wall. This concentration in a diseased wall is higher than the concentration in a physiologic arterial wall. For these reasons, in this work, we applied the plaque growth model in the normal, over- and under-estimated reconstructed geometries, and we compared all the calculated variables produced by the model, including the newly generated geometries, in terms of the simulated arterial wall area.

### 2.5. Error Propagation to the Prognostic Model

In this section, we present the results of the propagated error in the prognostic model of plaque growth prediction. The results of this model were previously presented [5]. Briefly, this predictive model is based on the computational modelling of blood flow, LDL transport and SmartFFR calculation, and these results are combined with morphological characteristics and non-imaging data to build binary logistic models of prediction of plaque progression. For the purposes of this analysis, we have to consider the following errors which can be propagated in the results of the prognostic model: (i) the error of the reconstruction, and (ii) the analytical error of the biohumoral data collection. Indeed, analytical errors in biochemistry are categorized into three main categories [16,17]. The pre-analytical error regards mainly the following types of errors: inappropriate testing, wrong patient identification, error during transport, wrong sampling and other. This error may range from 46–68.2%. During SMARTool clinical study such errors are not expected due to the many levels of security related to the pre-analytical step of data collection. The analytical error category refers mainly to issues with the proper function of the equipment, issues with quality control or incorrect procedures. This error may range from 7–13%. Still in our clinical study, we expect that this error is again minimized due to the experience of the clinical centers. However, in order to present this error, we assumed two different case scenarios for the error propagation study. In the first one, we apply the maximum error of 13% to all biohumoral variables, and in the second scenario we apply a random error with maximum of 13%. In the second case, the error may have a reducing or increasing effect to the feature value (each biohumoral value was multiplied by a random value ranging from 0.87–1.13). Finally, post-analytical error is due to the failure in reporting correctly the analysis results, and ranges from 18.5–47%. We assumed this error to be minimal because all values have been revised at least two times by the coordination team and because the SMARTool Case Report Form Application has specific rules to testify to the validity of the entered data.

## 3. Results

Simulations were performed to 20 arterial segments and to additional 40 segments which includes the propagated reconstruction error. A 5% reconstruction error was assumed, which was extracted during the validation of the reconstruction algorithms. This error was used to calculate the differences in the ESS distribution, as well as in the SmartFFR calculation, which refects a non-invasive diagnostic index for significant obstructive atherosclerotic disease.

### 3.1. Error Propagation to ESS

**Figure 2:**
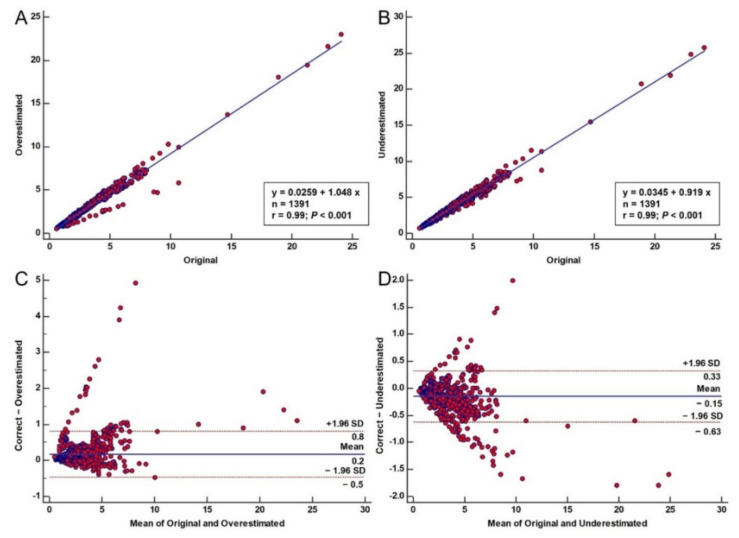
Error propagation to ESS: regression plot comparing the underestimated (**A**) and overestimated models (**B**) to the original ones. (**C**,**D**): Bland–Altman plots comparing the correct 3D models to the underestimated (**C**) and overestimated (**D**) ones.

To determine the effect of the error propagation on the ESS distribution, we created regression plots for the underestimated and overestimated 3D models for comparison to the correct 3D models for all cases. From the regression plots, we can see that both the underestimated (i.e., r = 0.99, *p* < 0.001) (Figure 2A) and the overestimated cases (i.e., r = 0.99, *p* < 0.001) (Figure 2B) are well correlated to the original 3D models. However, some points in the regression plots present a considerably different distribution. These points each regard the measurements of a specific vessel which produces these results may be caused by low vessel quality during the transformation, bad mesh quality or poor translation of the 2D contours. Moreover, we created Bland–Altman plots to understand the agreement of the overestimated and the underestimated models when compared to the correct 3D models. As depicted in Figure 2, good agreement was observed for most patients. However, as explained above, one patient exhibited significant differences which are also present in the Bland–Altman plots. Regarding the underestimated cases, we observed a mean difference of −0.149 ± 0.2430 Pa compared to the correct 3D cases (Figure 2C). The lower limit was −0.6262 (95% CI–0.6480 to −0.6043), while the upper limit was 0.3263 (95% CI 0.3044 to 0.3481). For the overestimated cases, we observed a mean difference of −0.1725 ± 0.3243 Pa compared to the correct 3D cases (Figure 2D). The lower limit was −0.4632 (95% CI −0.4923 to −0.4340), while the upper limit was 0.8082 (95% CI 0.7790 to 0.8373).

### 3.2. Error Propagation to SmartFFR

**Figure 3:**
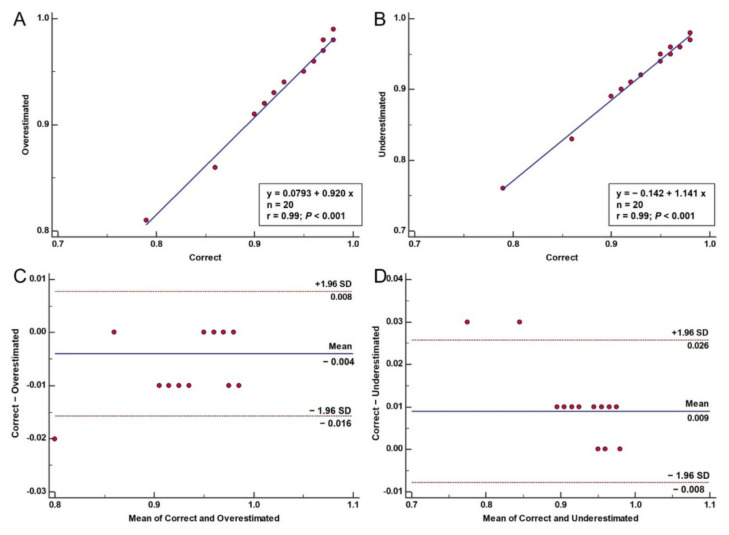
Error propagation to SmartFFR: Regression plots comparing the SmartFFR values of the overestimated (**A**) and underestimated (**B**) models to the original ones. Bland–Altman plots comparing the correct 3D models to the overestimated (**C**) and underestimated (**D**) ones.

Another important hemodynamic factor that was used in our error propagation study was the SmartFFR. We calculated SmartFFR in the 20 cases (original 3D models, underestimated and overestimated). Table 1 presents the calculated SmartFFR values for all 20 cases. We also created the respective regression plots comparing the overestimated and the underestimated models to the correct ones, as well as the respective Bland–Altman plots (Figure 3). From the regression plots, we can observe a strong correlation of both the overestimated (r = 0.99, *p* < 0.001) and the underestimated (r = 0.99, *p* < 0.001) models to the correct ones, respectively. Good agreement is also found between the overestimated (mean difference: −0.004 ± 0.006, *p* = 0.0075) and the underestimated models (mean difference: 0.009 ± 0.008, *p* = 0.0001) when compared to the correct 3D models. 

### 3.3. Error Propagation to Plaque Growth Prediction

The following parameters were used for the presentation of the error propagation results to the plaque growth modelling: LDL concentration, HDL concentration, oxidized LDL concentration, monocyte cell concentration, macrophage cell concentration, synthetic SMC concentration, contractile SMC concentration, collagen concentration, cytokine concentration, foam cell concentration, plaque volume and area of simulated thickened wall. It is worth mentioning here that any changes in these parameters due to geometry error were caused mainly by the change to the ESS distribution. In particular, a reduced lumen area (underestimation error) will cause an increase in the accumulation of LDL and subsequently increased inflammation and plaque growth rates. On the contrary, an increased lumen area (overestimation error) will result in reduced plaque growth rates due to low LDL accumulation in the arterial wall. The mathematical explanation of this situation is given by the Kedem–Katchalsky equations applied as boundary conditions at the endothelial membrane of the plaque growth model [8,18]. The minimum, maximum, mean and std. deviation for all variables are presented in Table 2. In comparison to the original model, Table 2 presents the resulting relative error as the percentage difference between the two models (over and underestimated). 

Additionally, the additive uncertainty was calculated for each level of simulation. The uncertainty of the first level (3D reconstruction) was 0.09. In the second level, the blood flow modelling and calculation of shear stress, there was a minor increase in the uncertainty compared to the standard deviation. Finally, the third level, the plaque growth model, was affected by both the 3D reconstruction and shear stress uncertainty.

Table 3 presents the results of a univariate analysis for the association of plaque progression with the original values of biohumoral data, with a 13% maximum error and with a random error between 7–13%. As we can see, non-significant differences were observed for most biohumoral variables. Major differences were observed for the alkaline and triglycerides, in which they were correlated with plaque progression in the case of the original values and the maximum error, while they were not correlated in the case of random error. On the other hand, leptin was not associated with plaque progression in the case of the original values and the maximum error, while it was correlated in the case of random error.

In order to examine these minor differences to the detection of the independent predictors of plaque progression, we implemented a multivariate model to examine the effects of the errors on the predictors included in the disease progression model (Table 4). The results demonstrate that there was no difference in the statistically significant predictors of disease progression. A difference was observed at the random error simulation where leptin was included in the model without being statistically significant. Overall, all cases demonstrate that the age and the baseline plaque burden are the independent predictors included in the model.

## 4. Discussion

We presented an error propagation study and its effect on the calculation of critical hemodynamic parameters, such as ESS and intracoronary pressures (SmartFFR). Twenty arterial segments were initially reconstructed in 3D using CTCA images, and for each case we created two additional models, one depicting an overestimation of the area of the arterial lumen and one depicting an underestimation, both by 5%. The calculation of the ESS values revealed similar trends for both cases, with a high correlation coefficient value for both cases (i.e., r = 0.99 for both cases). As expected, the underestimated models presented with higher ESS values, whereas the overestimated models presented with lower ESS values. Moreover, from the ESS distribution diagrams we observed that the underestimated models produces ESS values of greater percentage difference at the sites of maximum ESS values when compared to the correct 3D models, while the overestimated models produced ESS values closer to those of the correct 3D models. We also observed that SmartFFR was affected by the reconstruction error, without being affected to such an extent that it would produce a false classification of the examined case (i.e., healthy or ischemic). In particular, the only pathological case of our dataset was correctly classified as such by both the over- and the underestimated model. The healthy segments were correctly assessed as healthy by both the over- and underestimated models. The maximum relative error in the SmartFFR calculations did not exceed a percentage of 3.8%, a value that was not statistically significant.

The initial errors that might appear in the overall modelling procedure were mainly caused by the imaging formation issues that arose from the CTCA equipment itself [9]. Several artefacts may be present due to numerous technical reasons that might affect the procedure.

Moreover, we estimated the propagated error in a previously presented and validated plaque growth model. In that case, the over- and under-estimated geometries were also used for plaque growth modelling. The statistical analysis showed very good correlation between the original geometries and the included error. For this analysis we have calculated also the relative error, but also the propagated uncertainty from the 3D reconstruction level to the blood flow dynamics and finally to plaque growth results. There was minor increase in the shear stress variable and most of the plaque growth results. However, the few plaque growth variables which presented high standard deviation were affected also by the reconstruction error. The uncertainty was calculated using the addition or subtraction formula assuming that the reconstruction uncertainty was 0.09 and, for the rest of the variables, the standard deviation noted in Table 2 was utilized. We can conclude that the error introduced by the geometries affected specific variables, but the overall effect on the plaque growth was minor, because plaque growth is affected by the combination of many variables.

Finally, we also examined error propagation in a prognostic model. For this purpose, we introduced the analytical error in clinical biochemistry from biohumoral data collection. This error, according to literature data, may be as high as 13%. The maximum error was used in addition to a random value of error with a maximum of 13%. The results showed that minor differences were observed in the prognostic model with most of the predictors to remain to all case scenarios (reference model and error included models).

Our work has the limitation that it is based on a special CVD population characterized by a low risk to present disease progression. This may affect the interpretation of the error propagation results. Especially, the SmartFFR error would be considerable when more patients would be available with FFR values around the grey zone. In the same manner, the plaque growth results and especially the prediction of the disease progression would be more be affected when more pathological (in terms of arterial stenosis) cases are included, since the disease progression would be rapid. Furthermore, the 3D reconstruction process of the error-generated models underlies a risk of distorting the final surface of the lumen, due to the fact that the point cloud was created by using edited contours that had a distance of 0.5 mm between them. This might cause a slight distortion of the edited model which, in turn, may can cause severely affected ESS values among the base and edited models. This was observed in one case from the entire dataset and this was the cause of the points that exhibited a difference of >1 Pa between the base and the overestimated models, respectively.

## 5. Conclusions

In this work, we present for the first time to our knowledge an error propagation study and the effect that CTCA imaging acquisition and arterial reconstruction may have to computational models. In particular, we initially investigated the effect of reconstruction error to blood flow modelling and SmartFFR calculation. SmartFFR can be used as a non-invasive diagnostic index of obstructive disease. Our results demonstrated that the reconstruction error was indeed propagated to the calculation of SmartFFR, which may alter clinical decisions. In our patients, the error-included calculations still classified patients correctly. However, careful interpretation of the SmartFFR values is required for the grey zone around 0.80. We also examined the propagation of the reconstruction error to a plaque growth model. In that case, it was found that the effect was minor, possibly because the calculated plaque volume was affected by many factors independent of the arterial geometries. Finally, to address the effect of the propagated geometry error to a prognostic model, we also considered the potential analytical error during the collection and measurement of biological parameters. In this case, we found that the prognostic model exhibits similar accuracy and almost the same predictors, meaning that this model is generalized well.

## Figures and Tables

**Table 1:** SmartFFR values for the original, underestimated and overestimated 3D models of all 20 cases, respectively.

Case	Original	−5%	5%
Case 1	0.96	0.96	0.96
Case 2	0.95	0.94	0.95
Case 3	0.97	0.96	0.97
Case 4	0.9	0.89	0.91
Case 5	0.91	0.9	0.92
Case 6	0.95	0.95	0.95
Case 7	0.95	0.94	0.95
Case 8	0.92	0.91	0.93
Case 9	0.97	0.96	0.97
Case 10	0.96	0.96	0.96
Case 11	0.93	0.92	0.94
Case 12	0.98	0.97	0.98
Case 13	0.79	0.76	0.81
Case 14	0.96	0.96	0.96
Case 15	0.86	0.83	0.86
Case 16	0.98	0.98	0.99
Case 17	0.96	0.96	0.96
Case 18	0.96	0.95	0.96
Case 19	0.97	0.96	0.98
Case 20	0.98	0.97	0.98

**Table 2:** Descriptive statistics for the LDL concentration, HDL concentration, oxidized LDL concentration, monocyte cell concentration, macrophage cell concentration, synthetic SMC concentration, collagen concentration, cytokine concentration, foam cell concentration, plaque volume and area of simulated thickened wall for the normal, overestimated and underestimated geometries, respectively. The relative error and uncertainty are also presented. The relative error is defined as the percentage difference between the two models (over and underestimated).

	Minimum	Maximum	Mean	Std. Deviation	Relative Error Minimum	Relative Error Maximum	Relative Error Mean	Uncertainty
Shear stress	0.577	24.1	2.5663	1.8863				
Overestimated	0.495	23	2.3938	1.7576	−14.2	−4.56	−6.72	1.7599
Underestimated	0.63	25.7	2.7163	1.9902	9.19	6.64	5.84	1.9922
Thickened Wall	2.55 × 10^−6^	2.70 × 10^−5^	1.23 × 10^−5^	5.38 × 10^−6^				
Overestimated	2.68 × 10^−6^	2.84 × 10^−5^	1.27 × 10^−5^	5.49 × 10^−6^	5.10	5.19	2.74	1.7599
Underestimated	2.44 × 10^−6^	2.84 × 10^−5^	1.20 × 10^−5^	5.35 × 10^−6^	−4.31	5.19	−2.81	1.9922
LDL concentration	5.54 × 10^−5^	6.64 × 10^−4^	2.27 × 10^−4^	1.35 × 10^−4^				
Overestimated	5.54 × 10^−5^	6.54 × 10^−4^	2.22 × 10^−4^	1.33 × 10^−4^	0.00	−1.51	−1.85	1.7599
Underestimated	3.62 × 10^−5^	6.70 × 10^−4^	1.80 × 10^−4^	1.55 × 10^−4^	−34.7	0.90	−20.5	1.9922
HDL concentration	6.69 × 10^−4^	8.06 × 10^−4^	7.45 × 10^−4^	3.68 × 10^−5^				
Overestimated	6.69 × 10^−4^	8.08 × 10^−4^	7.45 × 10^−4^	3.70 × 10^−5^	0.00	0.25	5.02 × 10^−2^	1.7599
Underestimated	2.41 × 10^−4^	1.79 × 10^−3^	1.02 × 10^−3^	4.84 × 10^−4^	−64.0	1.22 × 10^2^	38.3	1.9922
Oxidized LDL concentration	8.20 × 10^−4^	1.41 × 10^−3^	1.16 × 10^−3^	1.81 × 10^−4^				
Overestimated	8.30 × 10^−4^	1.41 × 10^−3^	1.17 × 10^−3^	1.81 × 10^−4^	1.22	0.00	0.36	1.7599
Underestimated	7.68 × 10^−4^	1.45 × 10^−3^	1.16 × 10^−3^	2.38 × 10^−4^	−6.34	2.84	−0.73	1.9922
Monocyte cells concentration	1.32 × 10^−7^	1.40 × 10^9^	4.13 × 10^8^	2.59 × 10^8^				
Overestimated	1.32 × 10^−7^	1.41 × 10^9^	4.09 × 10^8^	2.57 × 10^8^	0.00	0.71	−0.93	2.57 × 10^8^
Underestimated	1.47 × 10^−7^	1.37 × 10^9^	4.13 × 10^8^	2.74 × 10^8^	11.4	−2.14	−9.77 × 10^−2^	2.74 × 10^8^
Macrophage cells concentration	3.40 × 10^9^	2.65 × 10^11^	8.85 × 10^10^	5.59 × 10^10^				
Overestimated	3.40 × 10^9^	2.62 × 10^11^	8.76 × 10^10^	5.53 × 10^10^	0.00	−1.13	−0.98	5.53 × 10^10^
Underestimated	3.55 × 10^9^	3.02 × 10^11^	8.81 × 10^10^	5.91 × 10^10^	4.41	14.0	−0.36	5.91 × 10^10^
Synthetic SMC concentration	1.00 × 10^−18^	4.83 × 10^5^	5.79 × 10^4^	9.01 × 10^4^				
Overestimated	1.00 × 10^−18^	4.80 × 10^5^	5.76 × 10^4^	8.90 × 10^4^	0.00	−0.62	−0.65	89073.87
Underestimated	1.01 × 10^−18^	8.19 × 10^5^	1.74 × 10^5^	1.56 × 10^5^	1.00	69.6	2.01 × 10^2^	155950.1
Collagen concentration	5.60 × 10^−26^	2.67 × 10^−2^	3.20 × 10^−3^	4.98 × 10^−3^				
Overestimated	5.60 × 10^−26^	2.65 × 10^−2^	3.18 × 10^−3^	4.92 × 10^−3^	0.00	−0.75	−0.69	1.7599
Underestimated	5.65 × 10^−26^	4.53 × 10^−2^	9.64 × 10^−3^	8.62 × 10^−3^	0.89	0.69	2.01 × 10^2^	1.9922
Cytokine concentration	6.00 × 10^−2^	1.00 × 10^1^	2.79	1.92				
Overestimated	5.99 × 10^−2^	1.01 × 10^1^	2.77	1.89	−0.17	1.00	−0.99	2.5852
Underestimated	6.70 × 10^−2^	1.49 × 10^1^	3.48	2.84	11.7	0.49	24.5	3.4721
Foam cells concentration	2.61 × 10^6^	4.88 × 10^8^	1.44 × 10^8^	1.02 × 10^8^				
Overestimated	2.61 × 10^6^	4.93 × 10^8^	1.42 × 10^8^	1.00 × 10^8^	0.00	1.02	−1.03	1.01 × 10^8^
Underestimated	2.92 × 10^6^	8.12 × 10^8^	1.79 × 10^8^	1.53 × 10^8^	11.9	66.4	25.1	1.53 × 10^8^
Plaque volume	4.54 × 10^17^	8.46 × 10^19^	2.49 × 10^19^	1.77 × 10^19^				
Overestimated	4.53 × 10^17^	8.55 × 10^19^	2.47 × 10^19^	1.75 × 10^19^	−0.17	1.01	−1.03	1.75 × 10^19^
Underestimated	5.07 × 10^17^	1.41 × 10^20^	3.12 × 10^19^	2.66 × 10^19^	11.7	66.6	25.1	2.66 × 10^19^

**Table 3:** Univariate analysis for the association of plaque progression with the original biohumoral data values, with a 13% maximum error and with a random error between 7–13%.

	Original Values		Maximum Error		Random Error (7–13%)	
Effect	Estimated Regression Coefficient (95% CI)	*p*-Value	Estimated Regression Coefficient (95% CI)	*p*-Value	Estimated Regression Coefficient (95% CI)	*p*-Value
Alanine	0.001 (−0.005 to 0.007)	0.6657	0.001 (−0.004 to 0.006)	0.6837	−0.002 (−0.005 to 0.002)	0.3209
Alkaline	0.004 (0.001 to 0.007)	0.0089	0.003 (0.001 to 0.006)	0.0114	0.001 (−0.001 to 0.002)	0.4390
Aspartate	0.002 (−0.004 to 0.009)	0.4557	0.002 (−0.004 to 0.007)	0.5079	0.000 (−0.003 to 0.003)	0.9561
Gamma-GT	0.000 (−0.003 to 0.003)	0.8390	0.000 (−0.002 to 0.003)	0.7756	0.000 (−0.002 to 0.002)	0.9581
Creatinine	0.174 (−0.111 to 0.459)	0.2302	0.108 (−0.119 to 0.335)	0.3479	−0.010 (−0.106 to 0.085)	0.8340
Uric acid	−0.015 (−0.059 to 0.028)	0.4893	−0.014 (−0.051 to 0.023)	0.4471	−0.003 (−0.018 to 0.012)	0.6832
Glucose	0.001 (−0.002 to 0.004)	0.5752	0.001 (−0.002 to 0.003)	0.7143	−0.000 (−0.001 to 0.001)	0.7337
Triglycerides	0.001 (0.000 to 0.002)	0.0417	0.001 (0.000 to 0.002)	0.0485	0.000 (−0.000 to 0.001)	0.5595
Cholesterol	0.000 (−0.001 to 0.001)	0.8951	0.000 (−0.001 to 0.001)	0.9709	−0.000 (−0.001 to 0.000)	0.4114
LDL	−0.000 (−0.002 to 0.001)	0.7338	−0.000 (−0.001 to 0.001)	0.6909	−0.000 (−0.001 to 0.000)	0.3698
HDL	−0.000 (−0.003 to 0.003)	0.9506	−0.000 (−0.003 to 0.002)	0.8915	−0.000 (−0.002 to 0.001)	0.5006
Reactive Protein	0.077 (−0.001 to 0.155)	0.0528	0.068 (−0.001 to 0.137)	0.0543	0.056 (−0.013 to 0.124)	0.1133
Interleukin-6	0.006 (−0.036 to 0.049)	0.7645	0.005 (−0.032 to 0.042)	0.7818	0.000 (−0.035 to 0.035)	0.9904
Leptin	−0.004 (−0.010 to 0.002)	0.2241	−0.003 (−0.009 to 0.002)	0.2127	−0.006 (−0.011 to −0.001)	0.0186
ICAM1	0.000 (−0.000 to 0.001)	0.5538	0.000 (−0.000 to 0.001)	0.5972	0.000 (−0.000 to 0.000)	0.6356
VCAM1	−0.000 (−0.001 to 0.000)	0.4961	−0.000 (−0.000 to 0.000)	0.4612	−0.000 (−0.000 to 0.000)	0.5804

**Table 4:** Results of multivariate linear regression.

Case	Effect	Estimated Regression Coefficient (95% CI)	*p*-Value
Original values	Age	0.010 (0.002 to 0.019)	0.0137
	Alkaline	0.002 (−0.001 to 0.005)	0.2627
	Triglycerides	0.001 (−0.001 to 0.002)	0.3456
	CE_18_3	0.001 (−0.003 to 0.004)	0.6840
	CE_20_3	0.004 (−0.009 to 0.017)	0.5224
	CE_20_4	0.000 (−0.003 to 0.004)	0.7970
	PS_38_6	−0.110 (−0.360 to 0.140)	0.3865
	Baseline plaque burden	−0.011 (−0.012 to −0.009)	<0.0001
	Min ESS	0.003 (−0.004 to 0.011)	0.3676
	Max LDL concentration	−57.466 (−726.391 to 611.460)	0.8656
	SmartFFR	−0.018 (−0.372 to 0.336)	0.9202
Maximum error	Age	0.011 (0.003 to 0.019)	0.0107
	Alkaline	0.002 (−0.001 to 0.005)	0.2372
	Triglycerides	0.000 (−0.001 to 0.002)	0.3528
	CE_18_3	0.001 (−0.003 to 0.004)	0.6368
	CE_20_3	0.005 (−0.008 to 0.018)	0.4598
	CE_20_4	0.000 (−0.003 to 0.004)	0.9221
	PS_38_6	−0.131 (−0.382 to 0.121)	0.3059
	Baseline plaque burden	−0.011 (−0.012 to −0.009)	<0.0001
	Min ESS	0.003 (−0.004 to 0.011)	0.3930
	Max LDL concentration	−99.142 (−770.228 to 571.943)	0.7709
	SmartFFR	−0.014 (−0.367 to 0.339)	0.9376
Random error (7–13%)	Age	0.012 (0.005 to 0.020)	0.0023
	Leptin	−0.005 (−0.010 to 0.001)	0.0902
	CE_18_3	0.001 (−0.003 to 0.004)	0.6437
	CE_20_3	0.008 (−0.004 to 0.020)	0.1809
	CE_20_4	−0.000 (−0.004 to 0.003)	0.9308
	PS_38_6	−0.156 (−0.399 to 0.087)	0.2069
	Baseline plaque burden	−0.011 (−0.013 to −0.010)	<0.0001
	Min ESS	0.004 (−0.003 to 0.011)	0.2923
	Max LDL concentration	−33.879 (−693.428 to 625.670)	0.9194
	SmartFFR	−0.021 (−0.372 to 0.329)	0.9039

## Data Availability

Not applicable.
